# Adhesion glycoprotein CD44 functions as an upstream regulator of a network connecting ERK, AKT and Hippo-YAP pathways in cancer progression

**DOI:** 10.18632/oncotarget.3095

**Published:** 2014-12-30

**Authors:** Shiyi Yu, Xiuxiu Cai, Chenxi Wu, Lele Wu, Yuzhi Wang, Yan Liu, Zhenghong Yu, Sheng Qin, Fei Ma, Jean Paul Thiery, Liming Chen

**Affiliations:** ^1^ The Key Laboratory of Developmental Genes and Human Disease, Ministry of Education, Institute of Life Science, Southeast University, Nanjing, P.R. China; ^2^ Department of Medical Oncology, Jinling Hospital, Nanjing, P.R. China; ^3^ Laboratory for Comparative Genomics and Bioinformatics & Jiangsu Key Laboratory for Biodiversity and Biotechnology, College of Life Science, Nanjing Normal University, Nanjing, China; ^4^ Cancer Science Institute, National University of Singapore, Singapore; ^5^ Institute of Molecular and Cell Biology, A*STAR, Singapore; ^6^ Department of Biochemistry, Yong Loo Lin School of Medicine, National University of Singapore, Singapore

**Keywords:** CD44, Oncogenesis, Signaling network

## Abstract

Targeted therapies are considered to be the future of cancer treatment. However, the mechanism through which intracellular signaling pathways coordinate to modulate oncogenesis remains to be elucidated. In this study, we describe a novel crosstalk among ERK, AKT and Hippo-YAP pathways, with CD44 as an upstream regulator. High cell density leads to activation of ERK and AKT but inactivation of YAP in cancer cells. CD44 modulates cell proliferation and cell cycle but not apoptosis. The expression and activity of cell cycle genes were cooperatively regulated by ERK, AKT and Hippo-YAP signaling pathways through CD44-mediated mechanisms. In addition, CD44 depletion abrogates cancer stem cell properties of tumor initiating cells. Taken together, we described a paradigm where CD44 functions as an upstream regulator sensing the extracellular environment to modulate ERK, AKT and Hippo-YAP pathways which cooperatively control downstream gene expression to modulate cell contact inhibition of proliferation, cell cycle progression and maintenance of tumor initiating cells. Our current study provides valuable information to design targeted therapeutic strategies in cancers.

## INTRODUCTION

Cancer remains a leading cause of morbidity and mortality in humans. Usually viewed as an evolutionary process, cancer results from the accumulation of somatic mutations in the progeny of a normal cell, leading to a selective growth advantage in the mutated cells and ultimately, uncontrolled proliferation [1, 2]. In recent decades, research has characterized the cellular and molecular events that enable the malignant transformation of cells [3] and several pathways are implicated to play important roles in oncogenesis. These include the well-characterized extracellular-signal-regulated kinase (ERK) and AKT pathways and the newly emerging Hippo-YAP pathway.

In ERK pathway, ERK is a downstream target of ERK kinase (MEK) which can be activated by Raf serine/threonine kinases [4]. The ERK signaling pathway is often up-regulated in human tumors and is a major determinant in the control of diverse cellular processes such as proliferation, survival, differentiation and motility [5]. In AKT pathway, AKT, also known as protein kinase B (PKB), is a downstream target of phosphatidylinositol (3,4,5) trisphosphate (PIP3) which is generated by activation of phosphatidylinositol 3-kinase (PI3K) and dephosphorylated by phosphatase and tensin homolog (PTEN) [6]. Phosphorylation of AKT promotes tumorigenesis via several oncogenic events, including apoptosis and cell proliferation [7]. In Hippo-YAP pathway, YAP is a downstream target of a core kinase cassette that consists of mammalian STE20-like protein kinase 1/2 (MST1/2), large tumour suppressor 1/2 (LATS1/2), Salvador homologue 1 (SAV1) and MOB kinase activator 1A/B (MOB1A/B) [8]. The Hippo-YAP pathway, an evolutionarily conserved pathway, plays fundamental roles in the control of different tissues during development and regeneration as well as in cancer development [8, 9]. The Hippo-YAP pathway primarily acts by inhibiting the nuclear function of YAP to control downstream gene expression [10, 11]. During development and regeneration, YAP is reported to be a critical modulator during epidermal cell proliferation [10, 12]. In cancers, the abnormal activation of YAP causes ectopic cell proliferation [8] and was reported to be associated with many cancer types [8, 9, 11, 13, 14].

Signaling by the AKT and ERK pathways can collaborate to maintain cell viability [15]. AKT can phosphorylate YAP and lead to the nuclear export of YAP via its interaction with 14-3-3 which belongs to a highly conserved acidic protein family and functions as a adaptor and scaffolding protein to regulate many cellular processes including apoptosis and cell-cycle control [16, 17]. Recently, cooperation between AKT and the Hippo-YAP signaling pathways was proposed to control cell proliferation in response to extracellular cues via PTEN [18], and YAP is able to induce ERK and AKT phosphorylation [19, 20]. However, whether and how ERK, AKT and the Hippo-YAP pathways do cooperatively contribute to oncogenesis has not been addressed.

The cessation of proliferation due to contact inhibition at high cell density, a fundamental property of normal cells, is defective in cancer cells [21]. Abrogation of contact inhibition can be achieved by activating oncogenic pathways and/or inactivating tumor suppressive pathways. However, what pathways and how these pathways cooperatively contribute to the failure of contact inhibition in cancer cells is not clear.

CD44 is a ubiquitously expressed cell-surface proteoglycan that mediates cell-cell and cell-ECM interactions [22]. CD44 is highly expressed in tumor initiating cells and regarded as a cancer stem cell maker of almost all solid cancers, such as breast cancer [23], colon cancer [24], pancreatic cancer [25], non-small cell-lung carcinoma [26], head and neck cancer [27], bladder cancer [28], ovarian cancer [29], prostate cancer [30], cervical cancer [31], gastric cancer [32], hepatocellular carcinoma [33], human nasopharyngeal carcinoma [34] and melanoma [35]. Thus, it is not surprising that CD44 has surfaced as a potential therapeutic target [36]. However, the molecular mechanism of its involvement in these cancers is unclear. Previous studies have provided evidence to suggest that CD44 is an important regulator in signal transduction processes controlling cell proliferation, survival and differentiation [37]. Indeed, studies show that CD44 can activate AKT [7, 38-42], ERK [43, 44] and attenuate Hippo signaling to lead to YAP activation [45]. Although these studies have raised the exciting possibility that CD44 might be a common upstream regulator of the AKT, ERK and Hippo-YAP pathways, each of these findings was made by applying different treatments on different cell types. The oncogenic function of CD44 has yet to be fully established, since CD44 can also act as a tumor suppressor [46].

In this study, we define an ERK/AKT/Hippo-YAP regulatory network with novel intersects that integrate these key pathways in oncogenesis. This study further supports the complexity of these signaling networks in oncogenesis and provides valuable indications for developing targeted therapies.

## RESULTS

### Signaling network crosstalk among the ERK, AKT and Hippo pathways

**Figure 1:**
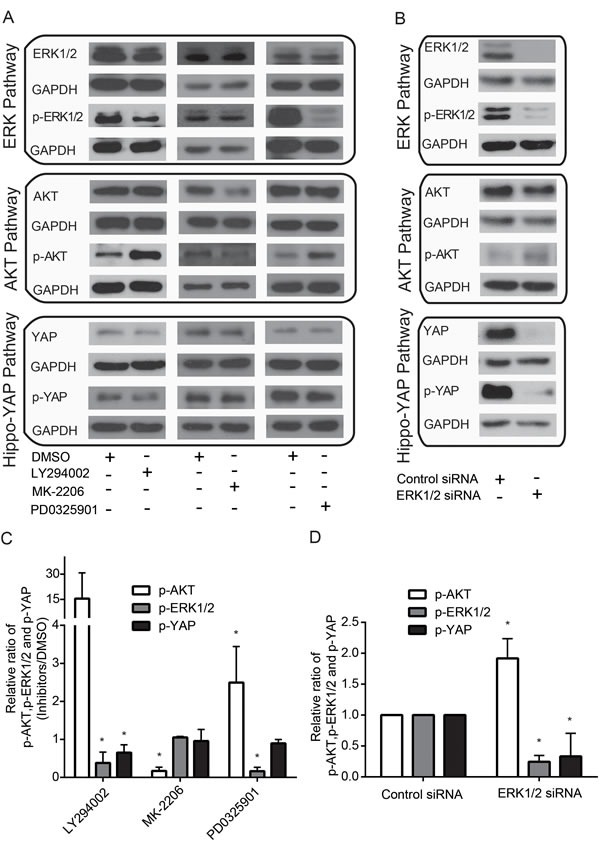
Crosstalk among ERK, AKT and Hippo-YAP pathways (A) Effects of PI3K inhibitor, LY294002, AKT inhibitor, MK-2206, and ERK inhibitor, PD0325901, on AKT, ERK and Hippo-YAP pathways. (B) Effects of ERK silencing on AKT, ERK and Hippo-YAP pathway. (C) Quantification of p-AKT, p-ERK and p-YAP upon treatment with LY294002, MK-2206 and PD0325901. (D) Quantification of p-AKT, p-ERK and p-YAP upon ERK silencing.

**Figure 2:**
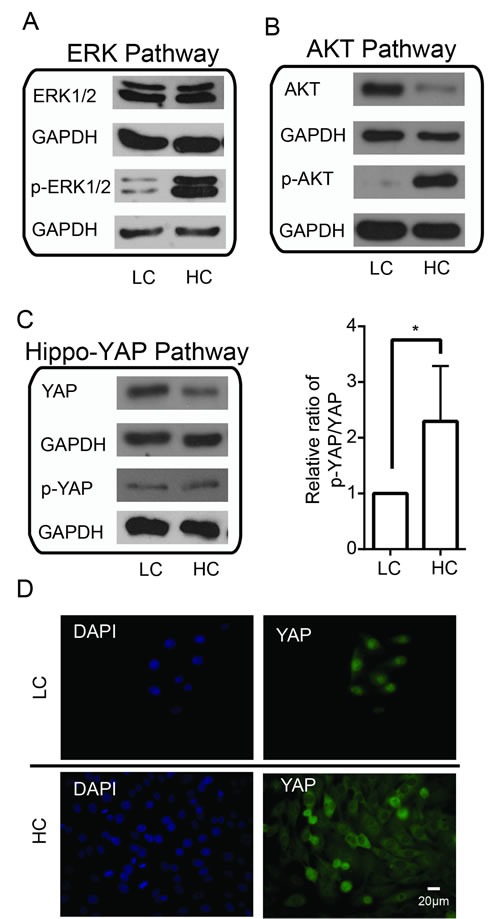
Alterations to ERK, AKT and Hippo-YAP pathways with respect to cell density High cell density induces activation of the (A) ERK pathway and (B) AKT pathway but inactivation of the (C) YAP pathway. (D) High cell density induces YAP nuclear-to-cytoplasmic translocation. LC, low cell density; HC, high cell density.

ERK and AKT pathways can collaborate to maintain cell viability [15]. However, the exact intersects between the ERK and AKT pathways are not fully understood. Here, we unexpectedly found that MDA-MB-435s cells treated with the ERK1/2 inhibitor, PD0325901, showed an increase in p-AKT, whereas treatment with the AKT inhibitor, MK-2206, did not significantly affect p-ERK1/2 levels (Figure 1A & C). Consistently, silencing ERK1/2 led to an increase in p-AKT levels (Figure 2B & D). Furthermore, treatment with LY294002, an inhibitor of PI3K—a well-characterized, major regulator of AKT activation—decreased p-ERK1/2 but increased p-AKT (Figure 1A & C). Studies have reported that YAP can induce ERK and AKT phosphorylation [19, 20] and that AKT can phosphorylate YAP [16]. However, it remains unknown whether ERK can modulate this YAP phosphorylation. Thus, we sought to test this again using ERK1/2 inhibition. We found a decrease in p-YAP upon treatment with PD0325901 (Figure 1A & C) and, consistently, found that silencing ERK1/2 with siRNAs also decreased p-YAP (Figure 1B & D). Likewise, treatment with LY294002 caused a decrease in p-YAP levels (Figure 1A & C), whereas treatment with the AKT inhibitor, MK-2206, which reduced both AKT and p-AKT levels, did not affect p-YAP (Figure 1A & C). Collectively, these finding suggest that ERK likely modulates both AKT and YAP activity, whereas AKT is unlikely to modulate the activity of both ERK and YAP.

### Involvement of ERK, AKT and Hippo-YAP pathways in cell contact inhibition

Cell contact inhibition is a fundamental property of a normal cell so that it ceases proliferating upon reaching confluence [21]. Failure in cell contact inhibition in cancer cells is known to contribute to tumorigenesis [47]; yet, the precise molecular mechanism is not clear. We therefore tested the expression and activity of these signaling molecules under high cell density conditions in MDA-MB-435s. We found that high cell density increased p-ERK1/2 without affecting total ERK1/2 levels (Figure 2A), indicating the activation of ERK pathway can be induced by high cell density. p-AKT levels were also increased but this was concomitant with decreased total AKT levels (Figure 2B), which suggests that high cell density can also induce the AKT pathway activation. Previous studies have shown that activation of Hippo-YAP pathway to phosphorylate and inactivate YAP was also linked with contact inhibition in MCF10A cultures [14]. We found that YAP expression decreased in high cell density conditions, with p-YAP remaining unchanged. Quantification of the ratio between p-YAP/total YAP showed an overall increase in p-YAP, thus indicating inactivation of YAP in the Hippo-YAP pathway (Figure 2C). Consistently, immunofluorescence staining showed that high cell density leads to the nuclear-to-cytoplasm translocation of YAP and its inactivation (Figure 2D). Taken together, these findings suggest that the ERK, AKT and Hippo-YAP pathways are involved in mediating contact inhibition, and that activation of oncogenic ERK and AKT pathways likely plays a major role in overcoming contact inhibition in cancer cells.

### CD44 modulates ERK, AKT and Hippo-YAP pathways in high cell density

**Figure 3:**
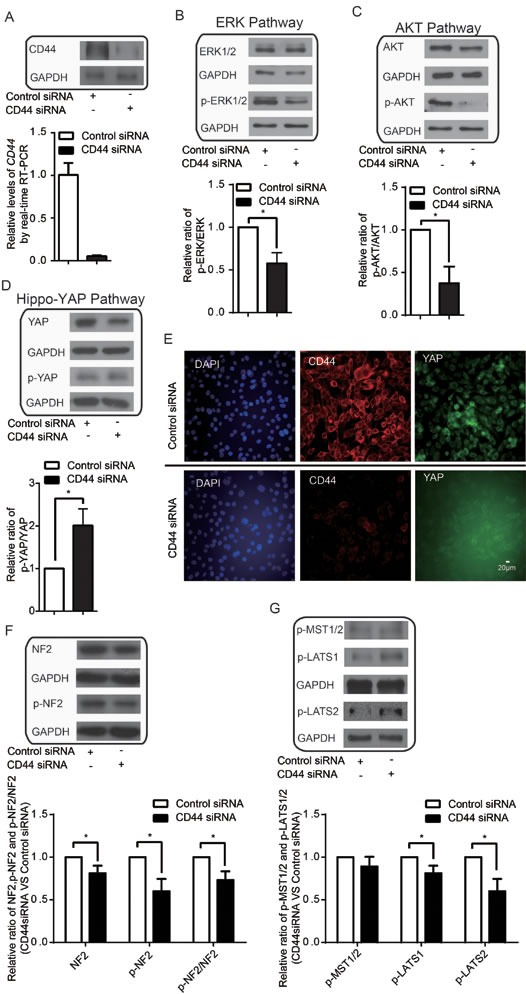
CD44 functions upstream of ERK, AKT and Hippo-YAP pathways (A) CD44 siRNA affects both mRNA and protein levels. (B-C) CD44 silencing inactivates the (B) ERK and (C)AKT pathways. (D-E) CD44 silencing inactivates YAP without affecting the nuclear-to-cytoplasmic translocation of YAP after the cells reach confluence. (F) CD44 silencing activates tumor suppressor function of NF2. (G) CD44 silencing activates tumor suppressor function of LATS1/2 not MST1/2. *p<0.05.

CD44 is involved in cell-cell and cell-matrix adhesion [48]. Previous findings that CD44 can activate ERK [43, 44] and AKT [7, 38-42] and attenuate Hippo signaling [45] collectively suggest that CD44 may function as a common upstream regulator of these three pathways to favor cellular avoidance of contact inhibition in cancer cells. To address whether this is indeed the case, we investigated the activity and expression of ERK, AKT and Hippo-YAP in conditions of high cell density with CD44 silencing in MDA-MB-435s and BT549. CD44 was successfully knocked-down by siRNA at both the mRNA and protein levels (Figure 3A & Figure S1A). We found that CD44 silencing led to a decrease in p-ERK1/2 and the proportion of p-ERK1/2 relative to total ERK1/2 protein (Figure 3B & Figure S1A-B). In addition, CD44 silencing led to a significant decrease in p-AKT and in the proportion of p-AKT relative to total AKT (Figure 3C & Figure S1A-B). CD44 silencing did not decrease AKT mRNA levels (Data not shown). These results indicate that CD44 senses the high cell density environment to activate AKT and ERK1/2, overall, suggests that depleted CD44 attenuates high cell density-induced activation of oncogenic ERK and AKT signaling pathways.

CD44 silencing also resulted in a decrease in total YAP protein, an increase in the proportion of p-YAP relative to total YAP (Figure 3D & Figure S1A-B), and a nuclear-to-cytoplasmic translocation of YAP in high cell density conditions (Figure 3E). Although high cell density still led to YAP inactivation in these cells (Figure 2D), the consistent effects on YAP with CD44 silencing and high cell density suggest that CD44 alleviates contact inhibition-induced inactivation of YAP to some extent; this occurs even though CD44 silencing could not reverse the high cell density-induced nuclear-to-cytoplasmic translocation of YAP.

**Figure 4:**
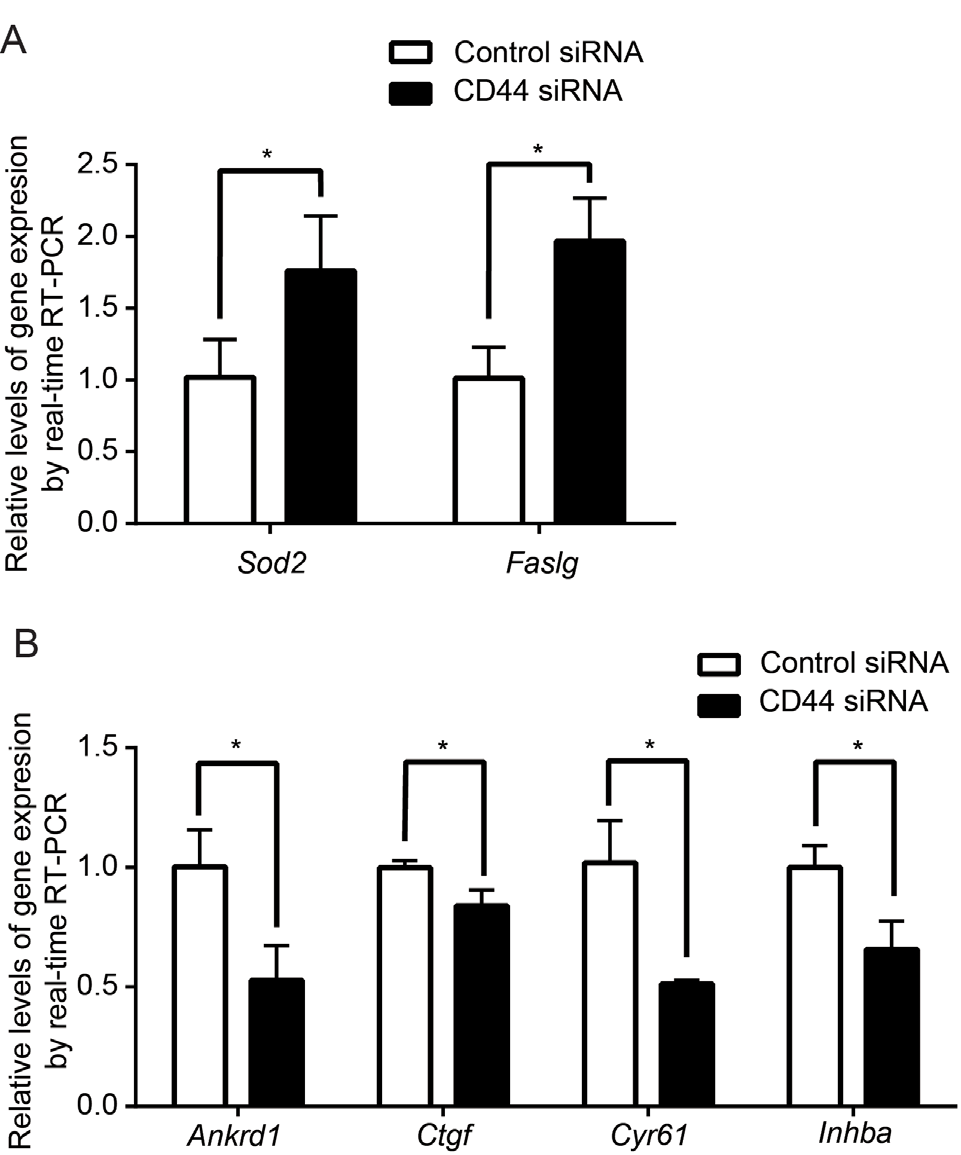
Effect of CD44 silencing on downstream target gene expression AKT downstream target genes, *Sod2* and *Faslg*, are upregulated with CD44 silencing (A) whereas Hippo-YAP downstream target genes, *Ankrd1*, *Ctgf*, *Cyr61* and *Inhab* are downregulated (B). *p<0.05.

To further confirm the upstream regulatory roles of CD44, we investigated the well-characterized downstream effector genes of the AKT and Hippo-YAP pathways in MDA-MB-435s. Consistent with our previous findings, Fas ligand (*Faslg*) and superoxide dismutase 2(*Sod2*)—both of which are AKT pathway target genes that are upregulated upon the suppression of AKT signaling [49-52]—were upregulated upon CD44 silencing (Figure 4A). YAP target genes, ankyrin repeat domain 1 (*Ankrd1*), connective tissue growth factor(*Crgf*), cysteine-rich angiogenic inducer 61 (*Cyr61*) and inhibin beta-a (*Inhba*) [19], by contrast, were downregulated upon CD44 silencing (Figure 4B). Taken together, these results suggest that CD44 functions as an upstream regulator of ERK, AKT and Hippo-YAP pathways and contributes to the failure of cell contact inhibition in cancer cells.

Neurofibromin 2 (NF2), also known as Merlin, was reported to mediate contact inhibition through interaction with CD44 [53, 54]. We found that CD44 depletion leads to decrease of p-NF2 and p-NF2/NF2, indicating the activation of the tumor suppressor function of NF2 (Figure 3F). CD44 depletion leads to increase of p-LATS1/2 but not p-MST1/2 (Figure 3G). These results together indicated that attenuation of high cell density-induced activation of ERK and AKT together with YAP inactivation by CD44 depletion might involved NF2 and LATS1/2 not MST1/2.

### Silencing CD44 inhibits proliferation and cell cycle progression

**Figure 5:**
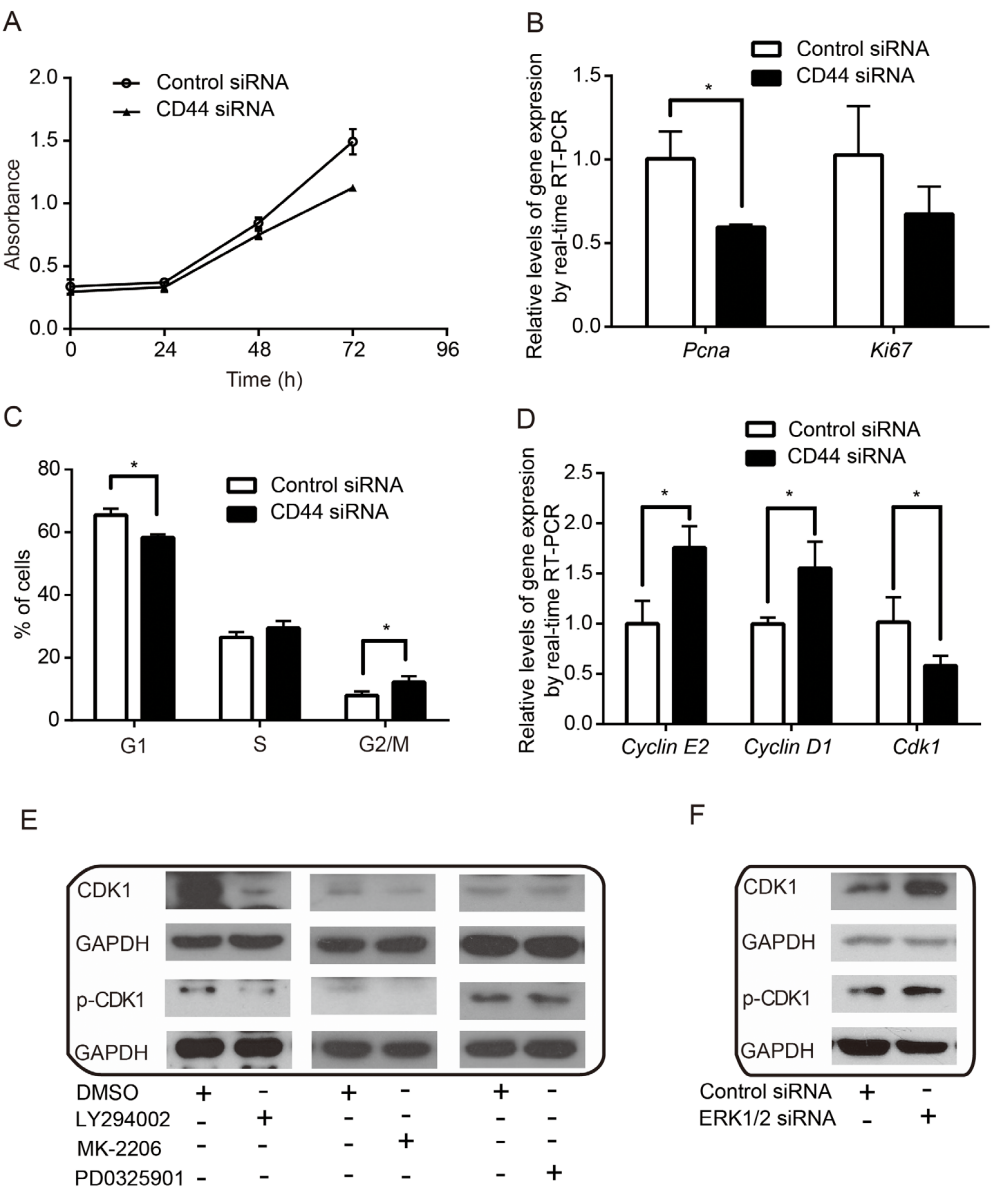
CD44 and its related signaling pathways are involved in cell proliferation and cell cycle control (A-B) CD44 silencing inhibits cell proliferation (A) and downregulates *PCNA* and *Ki67*(B). (C-D) CD44 modulates cell cycle progression (C) and the expression of key cell cycle genes, *Cyclin E2*, *Cyclin D1* and *Cdk1*(D). (E-F) Effects of PI3K inhibitor LY294002, AKT inhibitor MK-2206 and ERK inhibitor PD0325901 on CDK1 and its activity. *p<0.05.

CD44 is a transmembrane receptor associated with aggressive tumor growth, proliferation, and metastasis [55]. Although conflicting data implicate CD44 in both tumor suppression and tumor promotion [36], we found that silencing CD44 decreased the proliferation of cancer cells (Figure 5A & Figure S2). Consistently, the expression of proliferating marker genes, *Pcna* and *Ki67*, which are positively correlated with cell proliferation, was decreased upon CD44 silencing (Figure 5B). This may indicate that CD44 can promote cancer cell proliferation.

On the other hand, disruption of CD44 function was reported to induce apoptosis [56]. However, we found that CD44 silencing had little effect on apoptosis in cultured MDA-MB-435s cells with sufficient nutrition (Figure S3). Thus, we hypothesized that CD44 may contribute to cell cycle regulation to promote cancer cell proliferation. Indeed, following CD44 knockdown in MDA-MB-435s, we found that cancer cells tended to accumulate in the S- and G2/M-phases of the cell cycle and decreased the proportion of cells in the G1-phase (Figure 5C), alluding to a role for CD44 in cell cycle control. In particular, we found that the expression of key cell cycle genes, *Cyclin E2* and *Cyclin D1*, were increased, whereas *Cdk1* decreased at the mRNA level following CD44 inhibition (Figure 5D). Inhibition of PI3K and AKT also decreased CDK1 and p-CDK1, whereas ERK inhibition had little effect on the expression and of the phosphorylated protein (Figure 5E) but increased total CDK1 levels (Figure 5F). Our data support that proliferation and cell cycle progression are stimulated by CD44 and modulated through the cooperative activities of the downstream signaling networks.

### CD44 depletion abrogates cancer stem cell properties of tumor initiating cells

**Figure 6:**
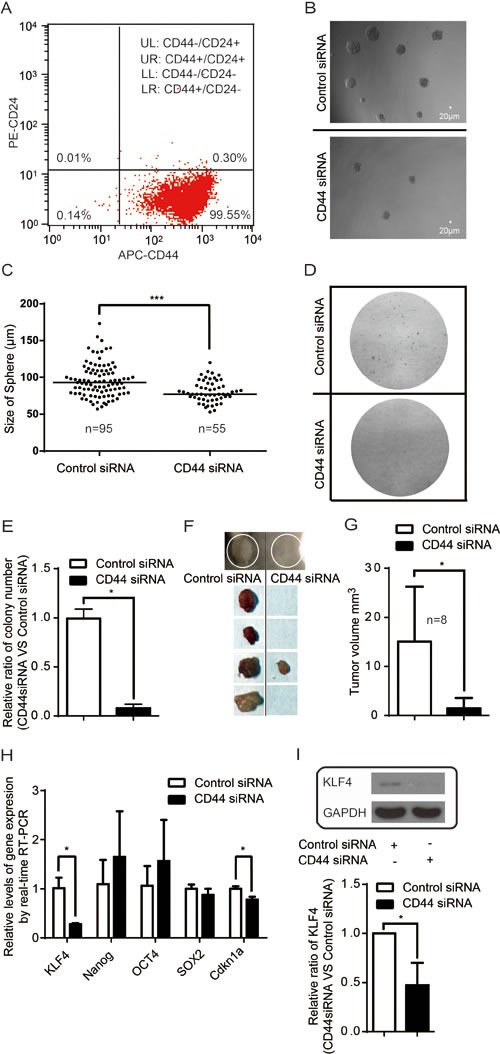
CD44 modulates stemness (A) MDA-MB-435s are almost all CD44+/CD24-(Low) cancer stem cell-like cells. (B) Inhibition of tumorsphere formation upon silencing CD44. (C) Quantification of tumorspheres (size >50μm) upon CD44 silencing. “n” represents the number of spheres with size >50μm formed amongst 1000 cells examined. ***p<0.0001. (D) Inhibition of colony formation upon silencing CD44. (E) Quantification analysis of number of colony forming in plate colony forming assay.*p<0.05. (F-G) Abrogation of tumor formation ability upon CD44 depletion in mouse model. *p<0.05 (H-I) Effects of silencing CD44 on the expression of key regulatory genes of stemness. *p<0.05.

Although no marker can be used universally to identify cancer stem cells, CD44 and CD24 are used extensively as potential surface markers with which to identify and isolate tumor initiating cells (cancer stem cells) in different cancers [57]. We found that more than 99% of MDA-MB-435s were gated as CD44^+^ or CD44^+^/CD24^Low^ (Figure S4 and Figure 6A), indicating that MDA-MB-435s cells are enriched of tumor initiating cells. Sphere-forming assays are widely used to identify stem cells and to evaluate the self-renewal and differentiation of tumor initiating cells [58]. In our sphere-forming assays, we show that CD44 silencing decreases the number and the size of tumorspheres (Figure 6B-C & Figure S5). Consistently, colony forming assay shows that the number of colonies was deceased upon CD44 silencing (Figure 6D & E). Tumor initiating cells are characterized by their ability to yield new tumors when xenografted into immunodeficient mice [59]. We found that silencing CD44 significantly reduced the tumourigenic potential of MDA-MB-435s in mouse model (Figure 6F & G).

Finally, KLF4, Nanog, OCT4, and Sox2 are known to be key regulators in maintaining the stemness of stem cells [60-62]. In MDA-MB-435s, we found that silencing CD44 caused a decrease in KLF4 expression (Figure 6H-I), which is required for the maintenance of the stem cell-like features of tumor initiating cells [63-65]. CD44 depletion leads to decrease of the expression of *Cdkn1a* (coding p21) (Figure 6H), a downstream target gene of KLF4 [66].

Thus, our findings may help to provide insight into the molecular mechanism of maintenance of tumor initiating cells.

## DISCUSSION

Over the past decades, there have been enormous efforts to study the molecular mechanisms controlling oncogenesis in order to identify targets to facilitate the development of directed therapies, an effort that is still regarded as the future of cancer treatment [67]. These efforts have led to the identification of many signaling pathways that play important roles in oncogenesis. ERK [5], AKT [5, 68] and the Hippo-YAP [8] pathways have all been found to play important roles in cancer development. However, how these pathways intersect and cooperate to contribute to oncogenesis and what common upstream regulator is involved in engaging this crosstalk to promote tumorigenesis is still unclear.

**Figure 7:**
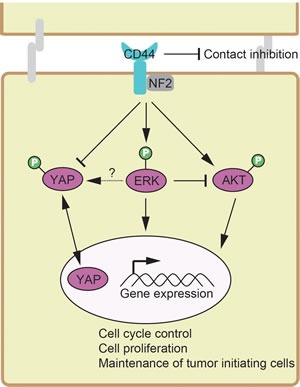
A paradigm showing the signaling network crosstalk among ERK, AKT and Hippo-YAP pathways, where CD44 functions as a common upstream regulator sensing cellular contact inhibition Positive regulation is shown as an arrow. Negative regulation is depicted as a blunt-ended line.

In current study, we describe here a novel cross-talk mechanism amongst ERK, AKT and Hippo-YAP pathways, which employs CD44 as a common upstream regulator to modulate signaling. AKT can inhibit ERK signaling and cause a shift in cancer cellular responses from cell cycle arrest to proliferation [69]. A recent study shows that PI3K inhibition—not AKT inhibition—causes the rapid inhibition of wild-type RAS and ERK pathway signaling [70]. Furthermore, it has been found that AKT re-activation is MAPK-ERK2-dependent [71]. Unexpectedly, AKT was activated upon inhibition of PI3K or ERK, whereas ERK was inactivated upon the inhibtion of PI3K rather than AKT (Figure 1). PI3K inhibition was found to down-regulates both the AKT and ERK pathways and AKT inhibition failed to block ERK pathway [72]. Inhibition of ERK pathway was reported to markedly enhanced phosphorylation of AKT (p-AKT) [73]. Taken together, the unexpected increase of p-AKT upon PI3K inhibition could be synergistic effects of PI3K and ERK. In addition, we found that ERK, AKT and the Hippo-YAP signaling pathways intersect to regulate each other and co-regulate downstream functions; this is in contrast to how they were originally modeled as linear signaling conduits (Figure 7).

Inhibition of the AKT pathway [74] and ERK pathway [75] greatly suppressed the expression of CD44. Silencing CD44 decreased p-ERK and p-AKT, indicating a decrease in ERK and AKT activation (Figure 3). These results suggest that CD44 and its downstream targets AKT and ERK might form the positive feedback loop. The Hippo-YAP pathway mediates the control of cell proliferation by contact inhibition [76]. Although the related mechanism is not fully understood, several upstream modules of the Hippo-YAP pathway, for example, adherens junctions, are claimed to sense the spatial and physical organization of cells in contact [76]. Our study implicates CD44 is one of these upstream modules of the Hippo-YAP pathway that senses cells in contact. The nuclear accumulation of YAP was reported as a novel downstream effect of PI3K, largely independent of AKT signaling [77]. Our finding that ERK inhibition/silencing not AKT inhibition decreases YAP phosphorylation led us to propose this new Hippo-YAP pathway sensing mechanism for contact inhibition involving CD44, PI3K and ERK but not AKT.

CD44 was reported to interact with NF2 to mediate contact inhibition [53, 54]. NF2, known as a tumor suppressor, is inactivated via phosphorylation [78-80]. It has been reported that that NF2 inhibits the activation of ERK and AKT pathways [81] and functions as upstream of Hippo-YAP pathway to activate Hippo-YAP pathway to inactivate YAP [54]. We found that CD44 depletion leads to decrease of p-NF2 and p-NF2/NF2, indicating the activation the tumor suppressor function of NF2 (Figure 3). Taken together, the inactivation of ERK, AKT and YAP upon CD44 silencing in high cell density might involve NF2. In the typical Hippo pathway, YAP was phosphorylated to be inactivated via Hippo core kinase cassette consisting of MST1/2 and LATS1/2 [14, 82-84]. Recently, AKT phosphorylation of MST2 and a feedback phosphorylation of Raf-1 by LATS1 was found to enable Raf-1 to suppress both MST2 and ERK signaling [85]. We found that CD44 depletion leads to increase of p-LATS1/2 but not p-MST1/2 (Figure 3). These results together indicated that LATS1/2 not MST1/2 were involved in attenuation of high cell density-induced activation of ERK and AKT together with YAP inactivation by CD44 depletion.

CD44 is critical in regulating cell adhesion, proliferation, growth, survival, motility, migration, angiogenesis, and differentiation [86]. We found that silencing CD44 inhibited cancer cell proliferation (Figure 5 & Figure S2). In addition, we found that CD44 was involved in cell cycle regulation and CD44 silencing increased the expression of *Cyclin D1* and -*E2* while decreasing the expression of *Cdk1* (Figure 5). Inactivation of AKT signaling inhibits proliferation and mediates G2/M phase arrest by downregulating *Cdk1* expression [87, 88]. Consistently, we found that inhibition of PI3K or AKT led to a decrease in CDK1 and p-CDK1 (Figure 5). Chiu and colleagues showed that ERK activation increased cell proliferation and caused an upregulation of CDK1 [89]. Interestingly, we found that inhibiting ERK activity using the inhibitor did not significantly change CDK and p-CDK, whereas silencing ERK led to a strong upregulation of CDK1 but not of p-CDK1 (Figure 5). YAP can be phosphorylated by CDK1 to promote mitotic defects, cell motility and neoplastic transformation [90]. These results together indicate that signaling pathways using CD44 as an upstream regulator function cooperatively to control downstream gene expression for cancer cell proliferation and cell cycle progression.

Silencing CD44 abrogates cancer stem cell properties of tumor initiating cells from decreased the number and size of tumorspheres and the number of colonies to tumourigenic potential (Figure 6). KLF4 is a key regulator of stemness [60-62] and stem cell-like features [63-65]. CD44 depletion decreased the expression of KLF4, Cdkn1a (coding p21) and Cyclin D1 (Figure 5-6). It was reported that *Klf4*-deficient mice express lower levels of p21, indicating that KLF4 positively regulates p21 gene expression [66]. P21 was found to suppress the expression of Cyclin D1 [91]. Taken these observations together, CD44-singaling networks-KLF4-p21-Cyclin D1 could explain, at least in part, the molecular events behind the involvement of CD44 in maintenance of tumor initiating cells. ERK1/2 signaling has been linked to regulating cell proliferation and cancer stem cell properties [92], and activation of the AKT pathway is deemed critical for the maintenance of EMT-associated cancer stem cell-like characterstics [74]. The Hippo-YAP pathway plays a critical role in stem cell [93] and cancer stem cells [94, 95]. Taken together, the paradigm described in current study is also valuable for understanding the molecular basis of maintenance of tumor initiating cells. We thus propose a signaling network with novel intersects (Figure 7), where CD44 senses of the degree of confluence to modulate a signaling network consisting of ERK, AKT and Hippo-YAP pathways, whereby it promotes the phosphorylation of AKT and ERK but inhibits the phosphorylation of YAP to activate oncogenic YAP activity. In conclusion, we described a paradigm in which through CD44, the signaling network consisting of AKT, ERK and Hippo-YAP pathways controls the expression of downstream genes that mediate contact inhibition, proliferation, cell cycle progression and maintenance of tumor initiating cells. The paradigm described in the current study deciphered the molecular mechanism underpinning oncogenesis at a signaling network perspective, and may suggest new targetable therapies for a directed therapeutic approach to cancer treatment.

## MATERIALS AND METHODS

### Cell culture and inhibitor treatments

MDA-MB-435s and BT549 were grown in Dulbecco's Modified Eagle's Media (Life Technologies; Carlsbad, CA) supplemented with 1% penicillin-streptomycin solution(Life Technologies), and 10% fetal bovine serum (HyClone; NY, USA). MDA-MB-435s were treated with one of the following inhibitors:10 μM of PI3K inhibitor, LY294002 (Cell Signaling Technology; Danvers, MA) 2μM of AKT inhibitor, MK2206 (Selleck Chemicals; Houston, TX), or 1μM of ERK1/2 inhibitor, PD0325901(Selleck Chemicals) for 24h and then subject to western blotting.

### RNA interference

ON-TARGET plus siRNA targeting CD44 (Dharmacon; Lafayette, CO) or siRNA mixtures consisting of three siRNAs against ERK1 and three siRNAs against ERK2 (Santa Cruz Biotechnology;Dallas, TX) or the non-targeting control siRNA (Dharmacon) was transfected into MBA-MB-435s and BT549 using Lipofectamine RNAi MAX(Invitrogen; Carlsbad, CA) according to the manufacturer's instructions.

### Western blot analysis

Mouse monoclonal antibody against CD44 and rabbit polyclonal antibody against YAP were purchased from Santa Cruz Biotechnology. Rabbit antibodies against AKT, p-MST1/2(T183, T180), p-LATS1(T1049), p-NF2(S518), NF2, p-AKT(S473), and p-YAP(S127), and a mouse monoclonal antibody against CDK1 were purchased from Cell Signaling Technology. A rabbit polyclonal antibody against p-CDK1(T14) was purchased from Signalway Antibody LLC. Mouse antibody against p-LATS2(S83) was from Abnova (Taipei city, Taiwan). A mouse monoclonal antibody against GAPDH was from Kangchen. Rabbit polyclonal antibody against KLF4 was purchased from Abgent (San Diego, CA, USA) Western blotting was carried out following the standard procedure. Briefly, protein lysates were separated by SDS-PAGE, transferred to PVDF membranes, and immunoblotted with the respective antibodies as indicated above and in the figures. Blots were developed with SuperSignal West Femto Maximum Sensitivity Substrate (Pierce/Thermo Scientiﬁc, Rockford, IL).

### Immunofluorescence

MDA-MB-435s were grown on glass slides in 24-well plates for 24 h. After washing the wells with PBS, the cells were treated with 4% paraformaldehyde for 30 min, then permeabilized and blocked with 0.1% Triton X-100 in 1% BSA for 1h at room temperature. Mouse monoclonal anti-CD44 (SantaCruz Biotechnology) and rabbit polyclonal anti-YAP (SantaCruz Biotechnology) were used as primary antibodies and Alexa Fluor 488 or Alexa Fluor 594-conjugated secondary antibodies (Invitrogen) were employed to detect fluorescence. The nuclei were stained with DAPI (Vector Laboratories, Cambridgeshire, UK). Representative images were captured using the Leica DM5000 B microscope (Leica Microsystems, Buffalo Grove, IL).

### Real-time RT-PCR

Extraction of RNA from cell lysates was performed using the RNeasy kit (Qiagen, Hilden, Germany)followed by cDNA synthesis using PrimeScript RT reagent kit (TaKaRa, Otsu, Shiga, Japan). The synthesized cDNA was analyzed by quantitative PCR using SYBR Premix Ex Taq (TaKaRa) in a Bio-Rad CFX96 Real-time PCR system (Hercules, CA). GAPDH was used as an internal control. The real-time PCR primer sequences are listed in Supplementary Table S1.

### Cell proliferation assay

Cell proliferation was measured using the CCK-8 kit (Dojindo Laboratories, Kumamoto, Japan). Approximately 5×10^3^cells were seeded into the wells of 96-well plates. After treatment with siRNA, cells were grown for 24h, 48h, 72h or 96h. To each well, 10μl CCK-8 solution was added and the cells incubated at 37°C for 2h. Absorbance was read at 450nm using a Bio-Rad iMark plate reader.

### Cell apoptosis assay and cell cycle assay

Cell apoptosis assay was performed using Annexin-V/Dead Cell Apoptosis Kit (Invitrogen) and analyzed on a BD FACSCalibur flow cytometer (BD Biosciences, Franklin Lakes, NJ). For the cell cycle assay, cells were harvested by trypsinization and fixed with 70% ethanol at 4°C overnight. Cells were then stained with propidium iodide and the cell cycle distribution was analyzed using a BD FACSCalibur flow cytometer (BD Biosciences).

### Flowcytometric analysis

PE mouse anti-human CD24, APC mouse anti-human CD44, PE mouse IgG2a κ Isotypecontrol and APC mouse IgG2b κ Isotype Control were purchased from BD Pharmingen (San Diego, CA). Cells were resuspended at 1×10^6^cells per 100 μl of sorting buffer (1×PBS containing 0.5% bovine serum albumin) and incubated with pre-conjugated CD44-APC and CD24-PE primary antibodies for 10 min at 4°C. Three control groups were established for the first sorting: (1) cells labeled with the isotype antibodies of the above two antibodies, (2) cells labeled with the anti-CD44-APC antibody and the isotype control antibody for CD24, and (3) cells labeled with the anti-CD24-PE antibody and the isotype control antibody for CD44. The cells were washed in 1×PBS and centrifuged at 800g for 2 min. For flow cytometric analysis, cells were resuspended in sorting buffer after incubation with the primary antibodies.

### Sphere formation and plate colony formation assay

MDA-MB-435s were transfected with either ON-TARGET CD44 siRNA or NON-TARGET control siRNA, separately, as previously described above. In sphere formation assay, cells seeded into the wells of a 96-well, ultra-low attachment plate (Corning, New York, NY). To each well, 100 cells were added inserum-free DMEM/F12 containing 20 ng/ml basic fibroblast growth factor (bFGF, PeproTech; St.Louis, MO), 20 ng/ml epidermal growth factor (EGF, PeproTech), ITS (insulin-transferrin-selenium; Sigma-Aldrich) with B27 (Gibco, Life Technologies)and cultured for 6 days. The size of the tumorspheres was measured and those with size over 50 μm were counted and analyzed.

In plate colony formation assay, cells were seeded into 6-well plate (500 cells/well) with DMEM containing 10% FBS (HyClone) and 1% PS (HyClone) and cultured for 8 days. The colonies were rinsed with PBS, fixed with 4% paraformaldehyde for 30 min, and stained with Giemsa stain (Leagene) for 30 min. The number of the colonies were counted and analyzed.

### *In vivo* mouse model

Female athymic STOCK-Foxn1^nu^/Nju 4-week-old mice were obtained from Model Animal Research Center of Nanjing University. Control siRNA and CD44 siRNA transfected MDA-MB-435s cells (2×10^6^cells) were subcutaneously injected into the left and right armpit of mice respectively. After 2 weeks of tumor growth, the tumor size was measured using a caliper and tumor volume was calculated by the following formula: Volume=0.5×Length×Wideth^2^. All of the experiments were conducted in accordance with the instructional standard guideline of Southeast University for animal experiments.

## SUPPLEMENTARY MATERIAL FIGURES AND TABLE



## Abbreviations

APC: Allophycocyanin
BSA: bovine serum albumin
CCK8: cell counting kit-8
CD24: cluster of differentiation 24
CD44: cluster of differentiation 44
CDK1: cyclin-dependent kinase 1
CDKN1A: Cyclin-Dependent Kinase Inhibitor 1A(p21)
DAPI: 4′,6-diamidino-2-phenylindole
DMEM: dulbecco's modified eagle medium
ECM: extracellular matrix
EGF: epidermal growth factor
EMT: epithelial-mesenchymal transition
ERK1/2: extracellular signal-regulated kinase1/2
FBS: fetal calf serum
ITS: insulin transferrin selenium
KLF4: krüppel-like factor 4
MAPK: mitogen-activated protein kinase
MEK: mitogen-activated protein kinase kinase
MOB1A/B: MOB kinase activator 1A/B
MST1/2: Mammalian Sterile20-like Kinase1/2
NF2: neurofibromin 2
OCT4: octamer-binding transcription factor 4
PCNA: proliferating cell nuclear antigen
PE: Phycoerythrin
PI3K: phosphatidylinositol 3-kinase
PIP3: phosphatidylinositol (3,4,5) trisphosphate
PKB: Protein kinase B
PS: Penicillin-Streptomycin
PTEN: phosphatase and tensin homolog
SAV1: salvador homologue 1
SOX2: sex determining region Y-box 2
YAP: yes-associated protein
