# Supplementary material for: Adhesion glycoprotein CD44 functions as an upstream regulator of a network connecting ERK, AKT and Hippo-YAP pathways in cancer progression
Source: Oncotarget. 2014 Dec 30;6(5):2951–65. doi: 10.18632/oncotarget.3095 (PMC4413630; doi:10.18632/oncotarget.3095)
Supplement: Supplementary file 1 [file oncotarget-06-2951-s001.pdf]

Adhesion glycoprotein CD44 functions as an upstream regulator of a network connecting ERK, AKT and Hippo-YAP pathways in cancer progression

Supplementary Material

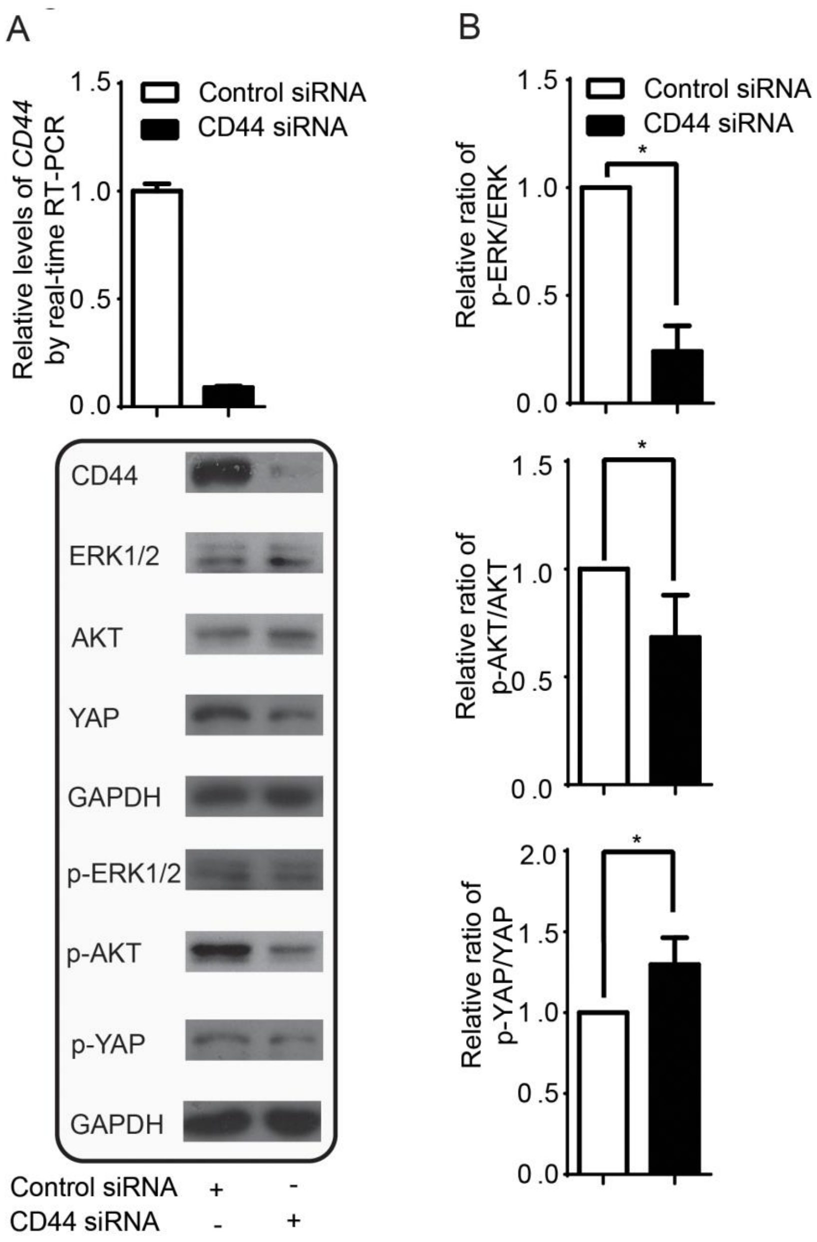

Figure S1: CD44 functions as an upstream regulator of AKT, ERK and Hippo-yap in BT549. * $p<0.05$

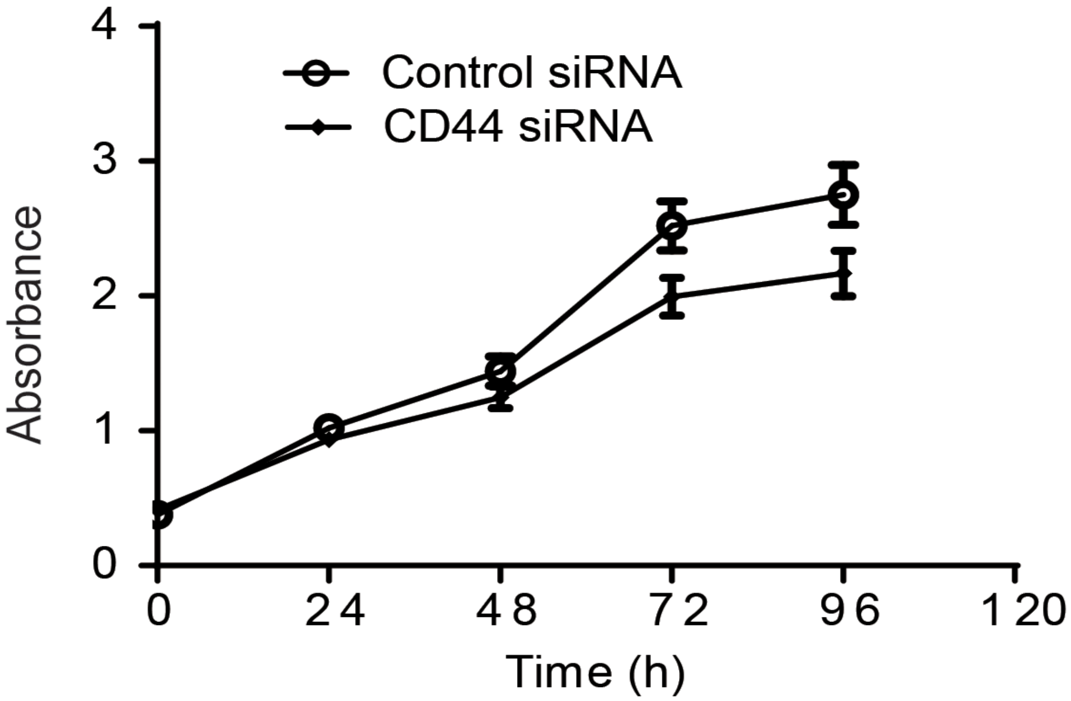

Figure S2: Silencing CD44 decreased the proliferation of BT549.

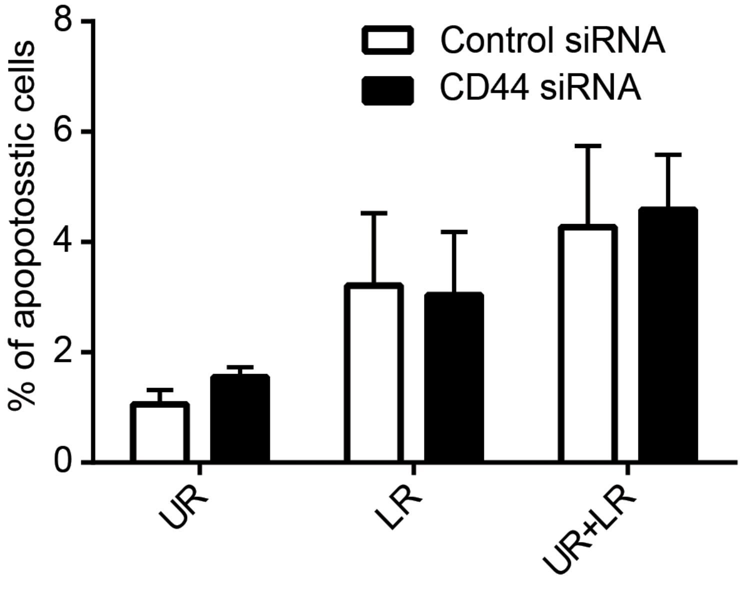

Figure S3: CD44 silencing had little effect on MDA-MB-435s cell apoptosis in cell culture with sufficient nutrition.

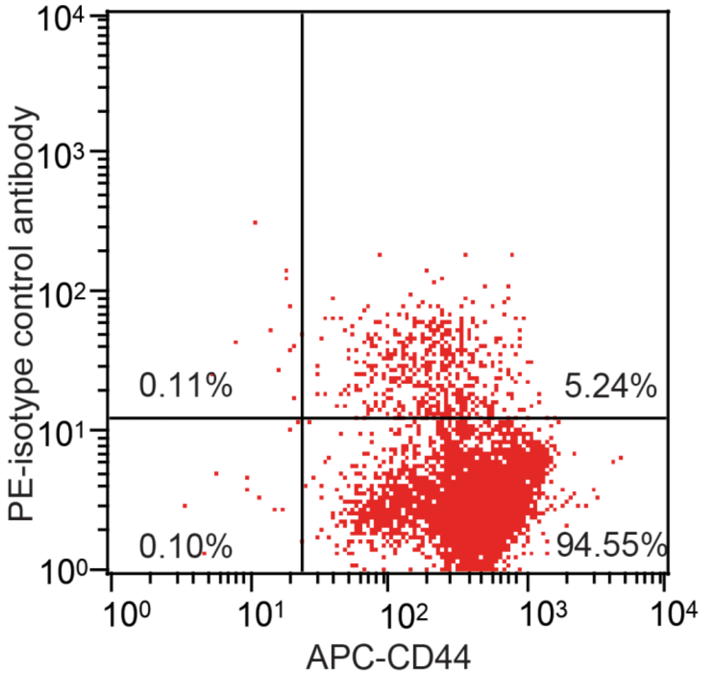

Figure S4: Almost all MDA-MB-435s cells are CD44^{+}.

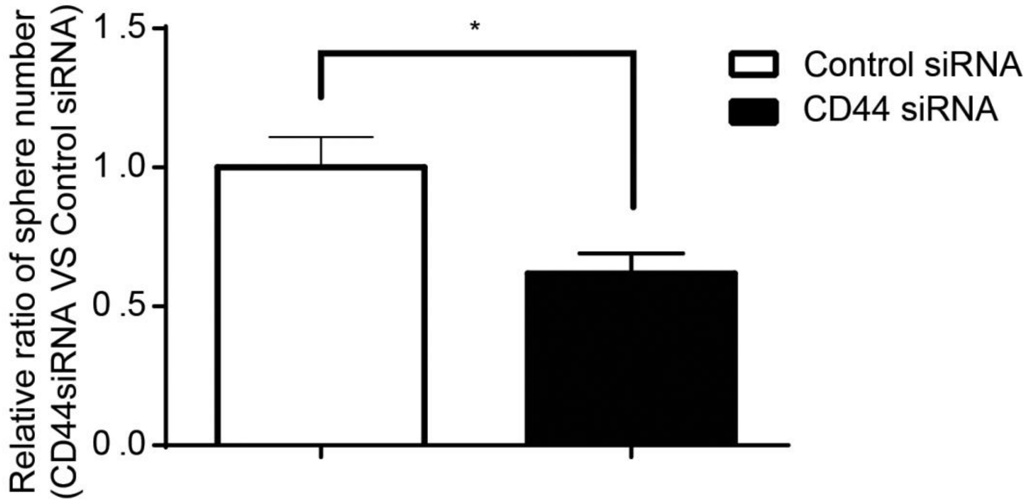

Figure S5: Silencing CD44 leads to decrease of tumorsphere number.

Supplementary Table 1.Real-time RT-PCR primers

Real-time RT-PCR primer	Sequence(5'-3')	Real-time RT-PCR primer	Sequence(5'-3')
Gapdh-RT-F	AGAAGGCTGGGGCTCATTTG	*CD44*-RT-F	GGTTACATCTTTTACACCTTTTCTAC
Gapdh-RT-R	AGGGGCCATCCACAGTCTTC	*CD44*-RT-R	GAATGTGTCTTGGTCTCTGGTAG
Pcna -RT-F	TGGAGAACTTGGAAATGGAAA	*Ki67*-RT-F	TCCTTTGGTGGGCACCTAAGACCTG
Pcna-RT-R	GAACTGGTTCATTCATCTCTATGG	*Ki67*-RT-R	TGATGGTTGAGGTCGTTCCTTGATG
Sod2-RT-F	TCCCAAGGGAAACACTCGGCTTT	*Faslg*-RT-F	GCAGCCCTTCAATTACCCAT
Sod2-RT-R	AAACCACTGGGTGACATCTACCAGA	*Faslg*-RT-R	CAGAGGTTGGACAGGGAAGAA
Inhba-RT-F	GCAGTCTGAAGACCACCCTC	*Cyr61*-RT-F	ACTTCATGGTCCCAGTGCTC
Inhba-RT-R	ATGATCCAGTCATTCCAGCC	*Cyr61*-RT-R	AAATCCGGGTTTCTTTCACA
Ankrd1-RT-F	AAGCAGGAGGATCTGAAGACACTT	*Ctgf*-RT-F	TGGAGATTTTGGGAGTACGG
Ankrd1-RT-R	GTTGTTTCTCGCTTTTCCACTGT	*Ctgf*-RT-R	CAGGCTAGAGAAGCAGAGCC
Akt1-RT-F	TGAAGGTGCCATCATTCTTG	*CyclinD1*-RT-F	GGCGGATTGGAAATGAACTT
Akt1-RT-R	ATGAGCGACGTGGCTATTGT	*CyclinD1*-RT-R	TCCTCTCCAAAATGCCAGAG
CyclinE2-RT-F	GCCAGACTTCTTTTGACATCCT	*Cdk1*-RT-F	TCCCTCCTGGTCAGTACATGG
CyclinE2-RT-R	TACAAGCTAAGCAGCAGCCC	*Cdk1*-RT-R	ACAAAACACAATCCCCTGTAGG
Nanog-RT-F	GGTGTGACGCAGAAGGCCTCA	*Sox2*- RT-F	GGGGAAAGTAGTTTGCTGCC
Nanog- RT-R	CCCAGTCGGGTTCACCAGGCA	*Sox2*- RT-R	CGCCGCCGATGATTGTTATT
Occt4- RT-F	GGCTCGAGAAGGATGTGGTCCG	*KLF4*- RT-F	CGAACCCACACAGGTGAGAA
Occt4- RT-R	GGGCTCCCATAGCCTGGGGT	*KLF4*- RT-R	GAGCGGGCGAATTTCCAT
Cdkn1a-RT-F	GGCAGACCAGCATGACAGATT		
Cdkn1a-RT-R	GCGGATTAGGGCTTCCTCT		
